# Factors that influence food choices in secondary school canteens: a qualitative study of pupil and staff perspectives

**DOI:** 10.3389/fpubh.2023.1227075

**Published:** 2023-07-14

**Authors:** Lauren D. Devine, Alison M. Gallagher, Stephen Briggs, Alyson J. Hill

**Affiliations:** ^1^Nutrition Innovation Centre for Food and Health, School of Biomedical Sciences, Ulster University, Coleraine, United Kingdom; ^2^Education Authority, Armagh, United Kingdom

**Keywords:** pupil, school staff, food choice, secondary school, canteen, adolescence

## Abstract

**Background:**

Adolescence is recognised as a period of nutritional vulnerability, with evidence indicating that United Kingdom adolescents have suboptimal dietary intakes with many failing to meet dietary recommendations. Additionally, adolescence is a time of transition when they become more independent in their dietary choices and begin to develop their own sense of autonomy and are less reliant on their parent’s guidance, which is reported to lead to less favourable dietary behaviours. Reducing the prevalence of poor dietary intakes and the associated negative health consequences among this population is a public health priority and schools represent an important setting to promote positive dietary behaviours. The aim of this school-based study was to explore the factors and barriers which influence food choices within the school canteen and to identify feasible strategies to promote positive dietary behaviours within this setting.

**Methods:**

Thirteen focus groups with 86 pupils in Year 8 (*n* = 37; aged 11–12 years) and Year 9 (*n* = 49; aged 12–13 years) in six secondary schools across Northern Ireland, United Kingdom were conducted. Additionally, one-to-one virtual interviews were conducted with 29 school staff [principals/vice-principals (*n* = 4); teachers (*n* = 17); and caterers (*n* = 7)] across 17 secondary schools and an Education Authority (EA) senior staff member (*n* = 1). Focus groups and interviews were audio-recorded, transcribed, and analysed following an inductive thematic approach.

**Results:**

Using the ecological framework, multiple factors were identified which influenced pupils’ selection of food in the school canteen at the individual (e.g., time/convenience), social (e.g., peer influence), physical (e.g., food/beverage placement), and macro environment (e.g., food provision) level. Suggestions for improvement of food choices were also identified at each ecological level: individual (e.g., rewards), social (e.g., pupil-led initiatives), physical (e.g., labelling), and macro environment (e.g., whole-school approaches).

**Conclusion:**

Low-cost and non-labour intensive practical strategies could be employed, including menu and labelling strategies, placement of foods, reviewing pricing policies and whole-school initiatives in developing future dietary interventions to positively enhance adolescents’ food choices in secondary schools.

## Introduction

Globally, adolescent overweight and obesity has increased significantly (1) and is now recognised as one of the most urgent public health challenges (2). This issue is particularly prevalent within the United Kingdom, with >30% of adolescents (aged 11–15 years) impacted by overweight or obesity (3). The negative physical (4, 5) and psychological (4–6) health implications associated with adolescent obesity are well-documented. Additionally, challenges also exist with reversing adolescent obesity, with 80% of obese adolescents likely to remain obese in adulthood (7), increasing the risk of further poor health outcomes in later life (8). Thus, determining effective preventative measures to mitigate the risk of obesity among this population is crucial to improve current and future health and minimise long-term obesity-related medical costs (9).

Less healthful dietary behaviours during adolescence, such as the overconsumption of energy-dense, nutrient-poor foods, can increase short (10) and long-term (11) obesity risk. United Kingdom adolescents’ dietary habits are of concern, with The National Diet and Nutrition Survey indicating suboptimal dietary behaviours among this population, including inadequate consumption of fruit and vegetables (12), low fibre intakes (13), excessive fat and sugar intakes (13) and higher energy intake also among those with overweight or obesity (14). As children transition to adolescents, they can become more susceptible to consuming an unbalanced diet (15) and dietary behaviours acquired during this period can persist into adult life (16). Therefore, dietary intervention during adolescence is essential to offset trends of declining dietary quality and establish healthy eating behaviours that can be sustained across the lifespan.

Adolescents are required to spend 190 days each year in school (17). Given the continuous contact time schools provide to this population, this setting represents a promising environment to deliver dietary interventions (18). School-based interventions are cost-effective (19) and offer the opportunity to reach the majority of adolescents, irrespective of socio-economic status or ethnical background (20). Moreover, adolescents’ consume a substantial proportion of their daily energy intakes in school (up to 1–2 meals per day) (21, 22). However, despite consistent efforts to determine the most effective school-based interventions to improve adolescents’ dietary intakes, outcomes remain short-term (23).

In Northern Ireland (NI), records suggest that more than half of adolescents (54–63%) typically consume school meals (provided by schools) at lunchtime (24) as opposed to a packed lunch (brought from home) or sourcing items from nearby food outlets. Mandatory food-based standards (25) are in place in NI schools to ensure pupils have access to a healthy and balanced school meal (26), which is of particular benefit to those who may have limited access to nutritious food outside school. However, although secondary schools provide healthier options compliant with the school-food standards, many adolescents continue to purchase the less nutritious items from the menu on offer (27), highlighting the need to explore alternate influential factors on adolescents’ lunchtime food choices. In addition to improved food provision, nutritional education is also compulsory in NI secondary schools (post-primary) for adolescents in Key Stage 3 (aged 11–14 years) (28), albeit, adolescents’ nutritional knowledge often has minimal impact on their food choices (29). Thus, identifying additional opportunities within the school-setting to promote positive dietary change is of importance. As pupils progress from primary to secondary education, parental control over their eating behaviours lessens and their propensity towards their dietary decisions become more independent-based (15). It is therefore pertinent to gain insight into the principal factors influencing adolescents’ school-based food choices as they develop increasing nutritional autonomy during this transitional period to optimise engagement and success of future school-based dietary interventions.

Research suggests that adolescents’ food choices within the school canteen can be influenced by various food-related factors, including available items, quality, appearance, taste, cost, value for money and peer pressure to opt for specific foods and canteen-related factors such as food hygiene, school menu and price displays, queue length and seating availability (30). More recent work has revealed adolescents’ favour take away items in the school canteen and associate ‘main meals’ as food to be consumed within the home environment (31).

In order to better understand the multiple levels of influence on adolescents’ food choices, Story et al. (32) proposed an ecological framework to consider their eating behaviours under four broad levels of influence to include individual (intrapersonal), social environmental (interpersonal), physical environmental, and macro level to aid in the design of appropriate nutrition interventions targeted at this population.

The difficulties associated with changing health behaviours are well recognised (33). When designing interventions, early involvement of stakeholders and the target user is recommended (34). In addition, although often under-utilised, qualitative research methodologies may assist in informing and optimising the design of interventions (35). Gaining further understanding of NI adolescents’ perspectives on the factors influencing their food choices within school and their suggestions on how best to address these factors through school-based strategies is needed if effective interventions to enhance positive dietary behaviours in this population are to be achieved. Additionally, a paucity of information exists on United Kingdom school staff’s perspectives on adolescents’ school-based food choices and their recommendations for improvement, limiting the ability for comparisons between key stakeholder groups to be examined. Furthermore, to aid in successful intervention design, consulting with school staff may provide researchers with a better understanding of any existing implementation practicalities to consider, such as schools’ academic priorities, available resources and the need to avoid over-burdening staff (36).

The aim of this study was to explore the primary factors influencing adolescents’ food choices within the school canteen environment from the pupil and school staff perspective. Additionally, a secondary aim was to identify feasible strategies to encourage healthful food choices amongst adolescents within the school-setting.

## Methods

### Study design

Qualitative research methods were selected to provide insight into the complexity of individuals’ food-related behaviours (37), in addition to their interactive nature to facilitate in-depth exploration of topics raised that is less possible with quantitative surveys (38, 39). Focus groups with pupils and one-to-one interviews with school staff were conducted to capture participants’ perspectives, attitudes, and experiences (40, 41). The reporting of this study is aligned with the consolidated criteria for reporting qualitative research (COREQ; Supplementary Table S1) (42). This study was conducted according to the guidelines laid down in the Declaration of Helsinki and all procedures involving human subjects were approved by Ulster University’s Research Ethics Committee (FCBMS-20-016-A; REC/20/0031). Written informed consent was obtained from all participants and their parents/ guardians.

### Sample selection and recruitment

#### School pupils

Year 8 (aged 11–12 years) and Year 9 (aged 12–13 years) pupils in seven purposively sampled (43) mixed-gender secondary schools in NI were invited to take part in this study. Year 8 (aged 11–12 years) and year 9 (aged 12–13 years) pupils were the focus as they had recently transitioned to secondary school and had become exposed to making independent food choices in the school canteen. Pupils who purchased food in the school canteen regularly (at least once each week) were eligible to participate. Schools were contacted via email or telephone and following agreement from the school principal, information sheets, assent and consent forms were distributed by a senior teacher to eligible pupils and asked to discuss with their parents/guardians. Participants who returned completed assent and consent forms were selected by a senior teacher to participate in the focus group.

#### School staff

A purposive sample (43) of school staff from a range of socio-economic (assessed using number of free school meals) and geographically diverse mixed-gender secondary schools (*n* = 17) across NI were invited to participate in this study. All grades of staff were eligible to participate including principals/vice principals and teaching staff from a range of subject disciplines. School catering staff included supervisors and caterers. Additionally, as the EA has responsibility for school meal provision in a large proportion of NI secondary schools, one senior EA staff member was invited to participate. School staff were contacted via email or telephone, and following agreement, information sheets and consent forms were distributed.

### Data collection

Pupils participated in mixed-gender focus groups and staff in one-to-one interviews, which were conducted independently by a researcher trained in qualitative research (L.D.D, PhD researcher, not affiliated with schools). Similar semi-structured discussion guides were used for the focus groups and interviews (Table 1) to ensure consistency and facilitate comparability between the pupils’ and staff’s perspectives. All focus groups and interview discussions were facilitated by the researcher using the topic guide to explore key issues. To enhance interaction and active listening during the discussions, notes were made directly after each session to enrich the data collected (40). Focus groups and interviews were undertaken until data saturation had been reached (44).

**Table 1:** Semi-structured discussion guide for pupils focus groups and school staff interviews.

Pupils focus group topic guide	School staff interview topic guide
Approximately, how many times a week would you eat school meals as opposed to taking a school lunch?	What is your opinion of the school meals provided in your school?
What is your opinion of the school meals provided in your school?	Do you feel any improvements could be made to the school meals provided in your school?
Do you feel any improvements could be made to the school meals provided in your school?	What is your opinion of the school canteen environment?
What is your opinion of the school canteen environment?	Do you feel any improvements could be made to your school canteen environment?
Do you feel any improvements could be made to your school canteen environment?	What do you perceive to be the most popular food options on your school lunch menu?
What are your favourite foods on the school lunch menu?	What do you perceive to be the least popular food options on your school lunch menu?
What are your least favourite foods on the school lunch menu?	What factors do you perceive to influence pupil’s food choice in the canteen at lunchtime?
What factors influence your food choice in the school canteen at lunchtime?	Are there any barriers that you perceive to prevent adolescents selecting the healthier food items in your canteen at lunch?
Are there any barriers that you perceive to prevent adolescents selecting the healthier food items in your canteen at lunch?	Are there any facilitators you perceive to encourage adolescents to select the healthier food items in your canteen at lunch?
Are there any facilitators you perceive to encourage adolescents to select the healthier food items in your canteen at lunch?	What changes, if any, to the school canteen environment do you perceive would encourage pupils to select healthier items at lunchtime that could be used as part of an intervention in school canteens?
What changes, if any, to the school canteen environment would encourage you to select healthier options at lunchtime?	Have you any additional thoughts, ideas or comments you would like to share that have not already been addressed in today’s discussion?
Have you any additional thoughts, ideas or comments you would like to share that have not already been addressed in today’s discussion?	

#### Pupils

Mixed-gender focus groups of 5–8 pupils were conducted between May and June 2021 within the school (classroom or hall) and during school hours under observation from a senior teacher. All pupils were reminded prior to commencing the focus groups that the information they provided would remain anonymous and would not be shared with their parents or school staff. The topic guide (Table 1) was designed and developed based on a review of the area and pilot tested on a small group of Year 8 pupils in different schools to test the questions for level of comprehension to optimise clarity of questions (45). Focus group sessions were on average 30 min duration (range 12–43 min).

#### Staff

One-to-one interviews were conducted remotely with school staff via Microsoft Teams or by telephone call at a suitable time for each participant between October and December 2020. Interview questions were pilot tested with one teacher in a different school to test suitability of questions. Interviews took on average 30 min (range 9–57 min).

### Data analysis

Focus groups and interviews were audio-recorded and transcribed verbatim. Transcripts were uploaded to NVivo 12 Pro Software (QSR International) for data management and analysed following the six phases of reflexive thematic analysis using an inductive approach (46). Codes were independently applied to quotes throughout each transcript by a member of the research team (L.D.D). To minimise the risk of bias and ensure correct interpretation of quotes, transcripts and codes were critically reviewed, discussed and confirmed by the research team (A.J.H and A.M.G). Quotes representing similar views were then clustered together and assigned initial sub-themes (L.D.D), which were reviewed by the research team (A.J.H and A.M.G) and refined before reaching consensus on the potential sub-themes. Each sub-theme was then mapped to each level of the ecological model, namely: individual (intrapersonal), social environment (interpersonal), physical environment, and macro environment (32). Quotes that were most reflective of the sub-themes were selected for inclusion.

## Results

### Participant and school characteristics

#### Pupils

Of the seven purposively sampled schools, six schools expressed an interest in participating and one did not respond to the study invitation. 86 pupils participated in 13 focus groups across the six schools (*n* = 4 urban; *n* = 2 rural) throughout three different district council areas in NI, with six focus groups undertaken with Year 8 pupils (*n* = 24 female; *n* = 13 males) and seven with Year 9 pupils (*n* = 28 female; *n* = 21 males). All six schools were co-educational and mixed-gender. Free school meal entitlement across the schools ranged from 21 to 53%.

#### School staff

Of the 35 participants who received initial invitations, 29 participated in this study (four did not respond to the study invitation; one did not return the consent form; one was excluded as they did not have recent experience in a secondary school). The final sample of 29 (24 females, 5 males) comprised principals (*n* = 2), vice-principals (*n* = 2), teachers (*n* = 17), catering staff (*n* = 7) sampled across 17 secondary schools, and a senior staff member (*n* = 1) in the EA. The schools were in urban (*n* = 14) and rural (*n* = 3) environments located within eight of 11 district council areas in NI. 16 schools were co-educational (mixed-gender) with one school being female only. Free school meal entitlement in these schools ranged from 7 to 54%.

The key sub-themes identified from the pupils’ and staff’s responses and exemplar quotes are reported in Tables 2, 3 using under the four levels of the ecological framework: individual (intrapersonal), social environment (interpersonal), physical environment, and macro environment (32).

**Table 2:** Pupils’ and school staff’s perceptions on the influences of adolescents’ dietary choices in the school canteen.

	Participant quotations	
	Pupil	School staff
Individual (intrapersonal)		
Internal motivations		
*Personal preference*	‘*I just pick what I like’* (P14, FG2, F) *‘It would be my choice for what I want to eat’* (P25, FG4, M)	*‘It’s down to personal preference for what they like and what they do not like’* (P9, T, F) *‘9 times out of 10, that age group are always going to go for what they prefer, and unfortunately what they prefer tends to be the less healthy options’* (P14, T, F)
*Taste*	‘*Because always unhealthy stuff tastes better, like yeah you want to get skinny or whatever, but oh well, it tastes nice so’* (P30, FG5, F)	*‘I think they are driven by what they like the taste of’* (P26, T, F)
*Appearance*	*‘It’s really all about appearance, appearance is a really big factor’* (P59, FG9, F)	*‘Quite often they do not want to try something because of the look of it perhaps’* (P17, T, F) *‘I truly believe kids keep with their eyes’* (P27, C, F)
*Habitual intake*	*‘I would just get the same thing every week because you know it’s nice’* (P78, FG12, M)	*‘A lot of them take the same thing nearly every day, they do not alternate at all’* (P1, T, F)
Time and convenience		
*Time restrictions and convenience*	*‘We just get panini’s and that’s it because it’s quicker…so then you have more time outside’* (P47, FG8, F) *‘It’s just really short, the lunches, you kind of just want to make the most of being outside before you go back to class’* (P14, FG2, F)	*‘I would say a huge factor is time and wanting to get outside rather than um actually sitting down to enjoy their meal’* (P22, T, F) *‘I think it’s things they can eat in their hands…they can carry outside with them’* (P8, T, F)
*Lunchtime activities*	*‘I sometimes just want to go outside to play’* (P63, FG10, M) *‘I would prefer to just grab and go, so I can go out and play football for longer with my friends’* (P10, FG2, M)	*‘Well, I do think particularly the boys like to get out to play football, so they will grab a panini and a juice carton and try to eat it quickly in the dining hall or slip out and eat it outside’* (P18, P, M)
*Queues*	‘*Sometimes if the queue is long, then I’d just get a drink and leave’* (P39, FG6, F) *‘If there’s big long queues, I just say forget about it and not eat’* (P79, FG11, M)	*‘They do not want to queue; they want to have their time’* (P20, C, F) *‘One factor I suppose would be the speed at which the dinner queue is moving*. *So, if there’s a short queue at the snack bar, they will go there just cause it’s quick and handy and they do not want to have to wait’* (P24, T, F)
Financial motivations		
*Price*	*‘The price can be a bit dear’* (P85, FG13, M) ‘*Not everyone can afford spending like £3 a day on lunch’* (P62, FG9, M)	*‘The pupils only have a certain amount of money to spend, that is a big thing with the students, it really is’* (P13, C, F)
*Value for money*	*‘Sometimes the money because for a little tiny pot, it could be like £2*.*20’* (P14, FG2, F)	*‘I think value for money is very important for them’* (P2, VP, F)
*Portion size*	*‘They have wee bowls of fruit, but like nobody ever takes them or the fruit pots because they are like, they do not fill you up’* (P20, FG4, F)	*‘Whenever they would go down to the canteen, it was sort of like the portion size that they would be looking at…so say if there was curry or something on, they’d be more inclined to go for that, not because they like the taste of it but because they’d think they were gonna get more food’* (P7, T, M)
*Nutritional knowledge*	*‘I do not mind if it’s healthy or not, it does not influence me’* (P17, FG3, M) *‘Do not really know if it’s healthy or not, I would just eat it’* (P32, FG5, M)	*‘Awareness does not correlate with the decisions they make’* (P8, T, F) ‘*They have the knowledge because they are taught it in class, so I know they do get it, they know what they should be taking, but knowledge is not enough to change behaviour*’ (P14, T, F).
Social environment (interpersonal)		
*Peer influence*	‘*Like today, one person got pizza and then everyone else just got the pizza, it’s just whatever everyone else gets, you have to get*’ (P22, FG 4, F) ‘*They usually just slag you and stuff for it, like they give you a bad name or something for like eating healthier’* (P10, FG2, M). *‘It’s not normal to eat healthy’* (P7, FG2, F)	*‘I would say it’s a lot to do with what their peers are eating*. *I do think a lot of them would just follow on from what others have chosen, maybe would not want to eat something different, just to be seen as different’* (P6, T, F) ‘*I also feel if one child lifts something to eat, a line of children want to eat the same thing, they go by what other pupils are eating you know*’ (P13, C, F).
*Home influence*	N/A	*‘If they are not eating the food at home, they are not going to eat it in school’* (P2, VP, F) ‘*If you are used to eating a certain diet at home, whether healthy or not, that will probably be mirrored in your choices that you make in the canteen’* (P12, T, M). *‘I think it very much, um, needs to be come from home as well*. *We can only do so much’* (P16, P, F)
Physical environmental		
*Placement of food and beverages*	*‘They keep all the fruit up in the corner, so you do not really see them, but like all the unhealthy foods, like the biscuits, are right beside the tills*. *The apples are sort of out of the way, so you would not pick an apple because the biscuits are there, so you would just pick a biscuit’* (P14, FG2, F).	‘W*hen a pupil comes in, it’s the first thing that they see is what they want’* (P13, C, F). *‘But there’s not enough of those [salads] sitting visually would be one fault’* (P10, T, M)
*Menu options and pricing information*	*‘You do not really know how much something is going to cost until you go to pay for it so you do not’* (P84, FG13, M) *‘Sometimes like, since you do not know what you are getting, if you like have not ate in the last couple of days or have only taken a bread roll, I do not want to have to do that again…cause you do not know what you are getting, you just take lunch instead’* (P2, FG1, F).	*‘They do not seem to read the menu to see what’s* on’ (P13, C, F) *‘I’d say most days pupils just walk past and then just decide whenever they get up to, up to the canteen’* (P6, T, F)
Macro environmental		
*Food provision*	*‘Whatever’s there, I’d just ate it’* (P83, FG13, M) *‘You just pick whatever is there like you do not really think about it’* (P14, FG2, F)	‘*First and foremost would be what is actually on offer will determine what they are eating’* (P21, T, F) *‘The variety of food on offer’* (P2, VP, F)

P, participant; FG, focus group; Y8, Year 8; Y9, Year 9; M, male; F, female; P, principal; VP, vice principal; T, teacher; C, caterer; and NA, not applicable.

**Table 3:** Pupils’ and school staff’s views on strategies to encourage selecting healthier options in the school canteen.

	Participant quotations	
	Pupil	School staff
Individual (intrapersonal)		
*Taster opportunities*	*‘Like if they gave us a wee taster’* (P6, FG1, F) ‘*They could give samples at the start of the day for new foods that would come in’* (FG11, P69, M)	‘*Maybe some kind of tasting type things where they get the opportunity to taste these vegetables without costing them money’* (P11, C, F)
*Autonomy in food choice*	*‘Even if there was a place where you can make up your own fruit box and add your own fruit because people might not like the grapes or the watermelon in it, so you could make your own fruit box’* (P72, FG11, F) ‘*I just like doing it myself’* (P21, FG4, F).	N/A
*Rewards and incentives*	*‘Maybe if you eat healthy, you could get 50% of your food’* (P8, FG3, M) *‘You could get like a reward card, like every time the dinner lady sees you get something healthy, they stamp it or something and then once you use up 5 [stamps], you get a free lunch or something like that’* (P68, FG8, F) *‘A VIP pass to the front of the line’* (P32, FG5, M)	*‘Even some sort of a rewards scheme that if you choose these meals, then you are put into a draw or something at the end of the month and you can win vouchers or something’* (P2, T, F) *‘Even if they had Like a loyalty card system, you know, like stamps’* (P26, T, F) *‘Pupils, especially Key Stage 3 pupils, really respond to rewards and achievement points’* (P5, T, F)
Social environment (interpersonal)		
*Pupil-led activities*	N/A	*‘I certainly think the younger pupils would be looking up to you know the older ones as role models and that could have a positive influence on them’* (P24, T, F) ‘*If you had those older ones maybe acting as healthy eating role models or prefects, that probably might encourage them*’ (P3, T, F).
Physical environmental		
*Labelling*	‘*Colours would help because if you say put the unhealthy options in maybe red, it would maybe drive us towards the green’* (P59, FG9, F) ‘*It would be nice if they could put like a green tick or something beside it to let you know what’s healthy’* (P20, FG4, F)	*‘The labelling of the food maybe could be better’* (P17, T, F) ‘*I think the traffic light system would be excellent’* (P1, T, F) *‘There could be more signage around the food and encourage them that way’* (P18, P, M) *‘I would maybe worry about focusing on the calorie content just for pupils who are maybe a little conscious of their weight’* (P5, T, F)
*Improved information accessibility*	*‘You’re kinda just guessing whether the food will be good or not that day… but if there was a menu, you could go ‘oh I do not like it that day’, so I could take a lunch and actually eat something instead of just taking a bread roll’* (P22, FG4, F)	*‘I do think maybe making their parents more aware of what kinda is available in the canteen would be something that would perhaps encourage them to choose healthier options…maybe putting on Facebook or giving them [pupils] a menu out to take home every week so that the parents knew what they were eating’* (P3, T, F)
*Placement manipulations*	*‘I would say show them more [healthier items] because they are hidden away in the fridge, like nobody ever sees them, we thought they were for the teachers, like nobody actually realised they are for us’* (P58, FG9, M) *‘Like the healthy stuff is way over there, but if they seen healthy stuff’s on offer, they’d be like, ‘oh yeah I will go get that, I forgot about that’…it could remind them that it is there’* (P54, FG9, M)	*‘The counter could be divided up into healthier options, you know, even start with the more healthier options and finish with less healthy options’* (P10, T, M) ‘*Try and change the positioning of it cause they have already chosen their foods before they get to the healthy options…so trying to have the first foods that they come to being the healthy ones’* (P6, T, F).
*Special offers*	*‘If the healthy foods were just really cheap that would drive people towards them because people go for the cheap’* (P59, FG9, F)	*‘Having some sort of special offers or something to sort of catch their eye on the healthier options…like a meal deal where they could get like a sandwich, the fruit and the drink, all for a better price’* (P6, T, F). *‘Possibly the healthier options a bit cheaper and advertised as that’* (P10, T, M)
Macro environment		
*Food provision*	*‘Not necessarily everything healthy, but a bit more healthy options’* (P16, FG2, M)	*‘Maybe take away the unhealthier options which is what we have tried to do, but the problem with that is then you will always get those who just walk past which is bad on two accounts*. *Because one, they are not getting anything to eat and 2, we are not getting the income’* (P11, C, F)
*Whole-school approach and educational practices*	N/A	‘*Maybe within certain subjects you know they could do activities on healthy eating*. *For example, for English, you could bring in like a healthy eating focus; maybe they could do a piece of persuasive writing to focus on the importance of choosing healthy foods’* (P5, T, F). *‘It would be a good idea to have at least one initiative, whole school, a year and try and drive that message home because in that way you are targeting the whole school population rather than just those in Home Economics’* (P8, T, F)

P, participant; FG, focus group; Y8, Year 8 pupil; Y9, Year 9 pupil; M, male; F, female; P, principal; VP, vice principal; T, teacher; C, caterer; and NA, not applicable.

### Influences on pupils’ food choices in the school canteen

#### Individual (intrapersonal)

Exemplar quotes to illustrate the following sub-themes are shown in Table 2.

##### Internal motivations

Pupils’ personal preferences, in addition to taste, appearance and habitual intakes, were important factors that influence their food choices in the school canteen. These factors often took precedence over the healthiness of the food items on offer, with many pupils commenting that options which were perceived to be less healthy were more tasteful. Pupils’ also commented that if they did not like the choices available, they may have not have lunch in the canteen that day.

*‘I just pick what I like’* (P14, FG2, F)

School staff had similar perceptions regarding personal preferences and reinforced that pupils were more likely to select items which were less healthy. School staff also reported that the appearance, familiarity and taste of the food were important when selecting items in the canteen and that these factors may act as barriers to pupils choosing alternate food options.

##### Time and convenience

Many pupils identified time restrictions as being a major barrier when making food choices as they have limited time for lunch break and many preferred convenient, ‘grab-and-go’ options as they preferred to maximise their free time with peers and participate in lunchtime activities. Additionally, the length of queue in the canteen was also commonly cited, with pupils’ opting for meals which had shorter queues, which may influence choice and possibly discourage pupils from eating in the canteen or skipping their lunchtime meal.

*‘If there’s big long queues, I just say forget it and not eat’* (P79, FG11, M)

This was similar to school staff’s views, who suggested that the queues were a factor which influenced food choices in the canteen and this issue was identified to be of greater importance for male pupils who prioritised socialising outside at lunchtime and were less likely to be waiting in queues. School staff also described how in more recent years, pupils’ choices have gradually changed over the years from selecting more traditional sit-down meals in the canteen to more convenient, portable and on-the-go options.

##### Financial motivations

Both pupils and staff commonly reported that price, value for money and portion size influenced food choice. For example, fruit options were reported to be of a small portion size and lower satiety value, thus, not good value for money, and therefore limited the selection of these items. Additionally, pupils reported that food items were expensive, with one pupil noting that on occasions, money allocated from ‘free school meal entitlement’ was insufficient to cover the cost of lunch. Financial motivations were identified as a key theme reported by five out of six schools regardless of whether the school was located in an area deemed to be rural or urban.

*‘Not everyone can afford spending like £3 a day on lunch’* (P62, FG9, M)

School staff also commented that they believed that pupils had a budget to purchase lunch and that the cost of food items and value for money was an important factor in their choice of food. It was noted by staff that dissatisfaction with food choice for value for money was perceived to increase the number of pupils opting for a packed lunch. School staff also commented that male pupils prioritise purchasing food which seemed to have larger portion sizes.

##### Nutritional knowledge

Pupils’ reported different views on the importance of understanding the nutritional value and composition of foods and whether the food was a healthy choice. Some pupils stated that they were unaware of which foods and meals were healthier, whereas, others were very aware of the healthier options. Both groups stated that this would not be a primary factor to influence their food choices.

*‘I do not mind if its healthy or not, it does not influence me’* (P17, FG3, M)

School staff agreed that they did not believe that nutritional knowledge was a key factor in influencing food choices of most pupils and reported that other factors, such as taste preferences, familiarity and convenience were more of a priority whilst in school. Nutrition education forms part of the curriculum for all secondary (post-primary) school pupils in NI for Year 8–10 (aged 11–14 years), however, staff reported that this knowledge was not considered to be sufficient to change their behaviour and translate into more positive health behaviours in the canteen.

#### Social environmental (interpersonal)

Exemplar quotes to illustrate the following sub-themes are shown in Table 2.

##### Peer influence

Peers were consistently identified as a major influence on pupils’ food choices. Pupils reported feeling pressurised to select similar items in the canteen to those of their peer group to avoid negative comments. Both male and female pupils expressed concerns about how their peers viewed them when making their individual food choices and that selecting certain food items in the canteen may not be considered socially acceptable.

*‘Like today, one person got pizza and then everyone else just got the pizza, it’s just what everyone else gets, you have to get’* (P22, FG4, F)

Peer influence was also the most dominant, reoccurring sub-theme within this level (social environmental) among school staff. School staff shared the view that pupils’ want to emulate their peers and aim to conform to what is perceived to be acceptable eating behaviours in an attempt to avoid standing out and to preserve a positive social status. In addition, catering staff reported viewing peer-induced choices in the canteen, with pupils selecting similar items to their friends.

##### Home influence

No pupils made reference to the influence of eating habits at home impacting on food choice in the school canteen. However, school staff expressed that eating habits established at home are reflective of pupils’ food behaviours in school and that both schools and parents need to promote positive eating behaviours to pupils simultaneously for the message to be impactful, as schools alone were considered to be insufficient to achieve sustainable positive dietary change.

#### Physical environmental

Exemplar quotes to illustrate the following sub-themes are shown in Table 2.

##### Placement of food and beverages

The location and ease of access to food and beverage items in the canteen was noted as being influential on food choice. Pupils’ acknowledged that healthier options were usually in a less prominent position in the canteen and often placed out of sight, having a direct influence on their purchasing decisions.

*‘The apples are sort of out of the way, so you would not pick an apple because the biscuits are there, so you would just pick a biscuit’* (P14, FG2, F)

School staff also recognised the impact of product placement on pupils’ food choice and cited that they are likely to opt for the food items which they observe first in the canteen.

##### Menu options and pricing information

Pupils indicated that they were often unaware of what foods were available on the menu in the canteen daily. This uncertainty of the menu was reported to impact on purchasing decisions, for example, pupils opting for a packed lunch or skipping their school meal. Pupils also noted dissatisfaction with clarity of pricing information and thus difficulties arose when choosing meals.

*‘You do not really know how much something is going to cost until you go to pay for it so you do not [buy it]’* (P84, FG13, M)

The majority of school staff members did not comment on school menus and pricing information in influencing adolescents’ food choice. A few school staff reported that lunchtime menus were displayed in their schools, however, considered them to be ineffective or overlooked by pupils.

#### Macro environment

Exemplar quotes to illustrate the following sub-theme are provided in Table 2.

##### Food provision

Pupils and school staff both cited food availability in the canteen as having a direct influence on the item’s pupils were consuming daily. Pupils also perceived there to be a lack of variety served in the canteen and that the options provided can often be repetitive. According to school staff, the canteen offered a good range of food options.

### Strategies to encourage selecting healthier options in the school canteen setting

#### Individual (intrapersonal)

Exemplar quotes to illustrate the following sub-themes are provided in Table 3.

##### Taster opportunities

To encourage the selection of healthier items in the school canteen, pupils’ and school staff recommended providing pupils with the option to sample certain food items prior to purchasing them to minimise financial risk.

##### Autonomy in food choice

Some pupils reported that combined food items in the canteen were off-putting, for example, mixed vegetable dishes and pre-made fruit salads. To counteract this barrier of improved food choices and to facilitate a higher uptake of these items in the canteen, pupils suggested that options be served separately to allow independent, self-selection of these items.

School staff did not directly comment on pupil’s autonomy to promote positive food decisions in the school canteen.

##### Rewards and incentives

The opportunity to receive rewards as a strategy to engage pupils in healthy eating practises in the canteen was a common, reoccurring sub-theme. Social rewards (e.g., trips, queue skips, extended lunchbreaks, sports activities, non-uniform day, and homework exemption pass), financial rewards (e.g., vouchers, discounted/free canteen items), and recognition rewards (e.g., certificates, awards/credit points) were reported as suitable incentives by pupils to encourage healthier choices in the school canteen. It was clear from the discussions that the concept of tracking their progress could stimulate further interaction with a reward scheme and incorporating in a competitive element at both individual and class group level.

*‘A VIP pass to the front of the line’* (P32, FG5, M)

School staff’s views reflected pupils’ in that they also recommended the use of social, financial, and recognition rewards to incentivise pupils to select healthier choices and considered that this would encourage pupils, in particular younger pupils, to be more proactive in their food-based decision making.

#### Social environmental (interpersonal)

Exemplar quotes to illustrate the following sub-theme are provided in Table 3.

##### Pupil-led initiatives

Pupils did not make suggestions on how their friends (e.g., pupil-led initiatives) could be a strategy for encouraging the selection of healthier items in the school canteen.

School staff recommended utilising peer networks as an effective means of facilitating positive food choices among adolescents and felt pupils were more likely to resonate with information provided by their peers than those delivered by staff. More specifically, school staff advocated for schools to implement specific roles for senior pupils to act as healthy eating ambassadors within the school to promote healthy eating.

#### Physical environmental

Exemplar quotes to illustrate the following sub-themes are provided in Table 3.

##### Labelling

When pupils were asked how best to promote selecting healthier options in the canteen, displaying nutritional labels was highlighted as a means of facilitating their ability to make informed decisions about food choices. Both male and female pupils recommended visual labelling schemes, for example, symbols or icons. Pupils also suggested that schools applying the traffic-light colour-coding system to food items in the canteen and to the school menus would be useful. In addition to nutritional labelling, pupils stated the importance of general food labelling, such as the food item name and ingredients.

*‘Colours would help because if you say put the unhealthy options maybe in red, it would maybe drive us towards the green’* (P59, FG9, F)

School staff also proposed labelling of foods and menus as an efficient strategy to facilitate positive food behaviours in the canteen. They suggested colour-coding and visual labelling, but urged the need for caution on calorie/energy labelling, stating concerns of the impact this may have on pupils who may already be weight conscious. School staff suggested that traffic-light labelling in particular would be applicable in the canteen setting and commented that pupils would be familiar with this scheme as it is covered early in the compulsory Home Economics Key Stage 3 (pupils aged 11–14 years) school curriculum. However, it was also noted that labelling schemes may be onerous on the canteen staff and adversely impact on their daily duties and should be considered.

##### Improved information accessibility

Another recommendation raised by the pupils was making menu and pricing information accessible in the canteen to enable them to make informed choices. Pupils suggested placing visual, eye-catching menu and pricing displays on the canteen walls that they could read readily whilst queueing. Additional menu and pricing information around the school, in classrooms, on school apps, websites and social media was also cited. Some pupils also advocated for healthy eating posters to be displayed in school, whilst others felt they would not be impactful. In addition, they expressed their desire to pre-order their meals, and to have knowledge in advance of what food items were going to be available daily.

School staff suggested further promotion of the school menus, for example, sending them to parents and uploading them on to the school apps, websites and social media platforms. School staff also recommended placing posters in different locations throughout the school.

##### Placement manipulations

Pupils and staff both commented on making improvements to where healthier food items were situated in the canteen. Pupils advocated for the healthier food items to be clearly visible in the canteen, whilst school staff advised ensuring that the healthier items were positioned more prominently and first in line in the serving area, as pupils can have their foods selected before having the opportunity to view the healthier options available.

##### Special offers

Applying special offers and reducing the price of the healthier items in the canteen was a view shared by both pupils and school staff. The concept of a healthy meal deal was reported amongst both pupils and school staff to allow pupils the opportunity to afford, for example, a healthy main meal, snack item and beverage.

*‘If the healthy foods were just really cheap that would drive people towards them because people go for the cheap’* (P59, FG9, F)

#### Macro environment

Exemplar quotes to illustrate the following sub-theme are provided in Table 3.

##### Food provision

Increasing the variety of foods on offer in the canteen was noted among pupils, with some pupils advocating for more healthy options, such as additional fruit and salad items.

School staff discussed restricting the sale of the less healthy options. However, it was also acknowledged that although the provision of healthy food items was a priority, it was important to recognise that the canteen was a business and there was a need to find a balance between serving the healthier options and those in demand by pupils in order to maintain pupil uptake and preserve the overall financial viability of the canteen.

##### Whole-school approach and educational practices

Pupils did not advocate for further input from school staff to encourage healthy eating or their nutrition-based education as strategies to promote improved food choices in the school canteen.

As Home Economics is a non-compulsory subject for pupils beyond Key Stage 3 education (pupils aged 11–14 years), principals and teachers suggested a whole-school, cross-curricular approach to delivering nutrition education to pupils which should involve other departments incorporating the promotion of healthy eating into their subjects. Some examples included the English department assigning pupils written tasks on the topic of healthy eating and the art department incorporating healthy eating poster campaigns as part of the curriculum. It was noted that as a start to have even one stand-alone lesson across a number of taught subjects would facilitate pupils thinking more about their food choices and could prove beneficial. School staff also acknowledged that healthy eating promotion should not be limited to the Home Economics department and suggested additional whole-school, educational strategies to promote healthy eating such as delivering talks during assemblies and educating parents.

*‘It would be a good idea to have at least one initiative, whole-school, a year and try and drive that message home, because in that way you’re targeting the whole-school population rather than those just in Home Economics’* (P8, T, F)

## Discussion

This study explored pupils’ (aged 11–13 years) and school staff’s perspectives on the main factors influencing adolescents’ canteen-based food choices and how best to encourage this population to select healthier food items within this environment. This qualitative research has identified several influential factors on food choices and recommended strategies for improvement to consider when designing future school-based interventions aiming to facilitate positive dietary behaviours among adolescents that are both acceptable to the target population and viewed as feasible for implementation by key school staff members.

In this present study, both pupils’ and school staff cited habitual intakes and personal preferences as important determinants of adolescents’ food choices within the school canteen. Food appearance and perceived taste were also identified as particularly salient factors, which can act as deterrents to selecting the healthier items available in the canteen. These findings support recent research by Glabska et al. (47) whereby adolescents considered sensory appeal of higher importance than health when determining food choices. Moreover, taste has consistently been identified in the literature to motivate adolescents’ school-based food choices irrespective of gender or location (30, 48–52). Opportunities to sample items served in the school canteen free of charge was a strategy proposed by participants in the present study, which aligns with previous United Kingdom research (53) reporting that 9–10-year-old advocated for exposure to new foods through school ‘taster sessions’.

The influence of queues on United Kingdom adolescents’ food choices in the school canteen has been reported from the early 2000s (38, 54). Results from the present study confirm that queue length remains a prominent factor when making dietary decisions in the school canteen. Data from the present study also identified that long queues can negatively influence adolescents’ food choices, and in line with previous studies in the United Kingdom (55) and further afield (56), can act as a barrier to school meal uptake, with pupils’ often sacrificing their school lunch due to long waiting times. Thus, efforts to alleviate the impact of queue length at lunchtime, such as implementing staggered breaks for different year groups or, as suggested in the present study, providing queue skips as a reward for healthy eating, may be measures for schools to consider to both encourage the uptake of school meals and establish healthier dietary habits within this environment. Findings from the current study also correspond with past research (57) that adolescents’ can be more inclined to opt for the grab-and-go options available in the canteen. As grab-and-go foods tend to be ultra-processed with typically high fat, sugar and salt contents (58), it is important that schools provide alternate nutritious, readily available grab-and-go options to support healthy school-based food choices.

In accordance with previous literature, highlighting the influential role of peer social conformity in influencing adolescents’ dietary intakes (59), the present study further evidences that peer acceptance is also fundamental when considering food options in the school-setting, which can impede the selection of healthier items. Recent work has highlighted gender differences among this age group, with females having larger concern for peer perceptions than males in relation to their dietary behaviours in school (60, 61). Interestingly, these gender disparities were not apparent in the present study, with both male and female pupils reporting feeling conscious and subject to disapproval from their peers if they opted for the healthier options, directly impacting their food choices in the school canteen. Given the perceived impact peers pose on purchasing decisions, school staff in the present study proposed involving peers in school-based strategies to promote positive dietary behaviours and particularly emphasised the opportunity to utilise senior peers in healthy eating school-based initiatives. In Australia, senior pupils have proven effective in role modelling healthful behaviours among the younger pupils in secondary school (62). Additionally, in America, a peer-led, school-based nutrition education intervention among adolescents was positively viewed and reported as feasible and acceptable among pupils, peer-leaders and teaching staff, with peer-leaders also citing improved dietary practices and awareness of healthy eating as a result of their role (63). However, the feasibility and acceptability of delivering a peer-led, school-based dietary intervention in secondary schools across NI is unknown, and thus, further investigation is warranted.

Whilst peers appear to have a significant role in influencing adolescents’ school-based dietary behaviours, school staff in the present study also cited the importance of home and parental influences in helping school’s shape adolescents’ dietary practices and were of the opinion that ‘*we can only do so much’*. This is comparable to an English study where secondary school headteachers and chairs of governors viewed parents as key influencers on adolescents’ lifestyle habits and that any measures taken by schools to improve these behaviours can either be supported or impaired by the home environment (64). Contrary to previous research (65), pupils’ in the present study did not acknowledge their parents or their home environment as an influential factor on their food choices in school, which may reflect how this population group place greater importance on expressing their own individual autonomy at this life stage. Future work to determine NI parents’/guardians’ perspectives on both their own and the school’s responsibilities in influencing adolescents’ food choices within the school-setting may be worthwhile.

Ultimately, participants in this study cited that adolescents’ food choices are influenced by what is available to them. In NI, assisted by a checklist, individual schools are required to self-monitor their compliance with the school food standards (66). In a recent study, NI stakeholders’ commented that adherence to the school-based standards may be negatively impacted due to a lack of monitoring (15), thus, implementation of a systematic monitoring process or procedure may be beneficial to enhance the provision of nutritious foods across NI schools.

Based on findings from the present study, pupils would welcome more frequent information on school meal choices on the menu and food prices, which would assist with pre-planning their meals. In addition to suggesting improvements to menu and pricing information, some pupils advocated for a pre-order lunch system, which has been previously shown to increase the selection of fruit, vegetable and low-fat milk items among youth in US school canteens, however, more research is required with larger sample sizes and in alternate locations to generalise these findings (67).

Incorporating labelling schemes for items served in the canteen was a leading strategy recommended from both pupils and school staff to improve food choices. Pupils and staff suggested visual labels, with pupils placing emphasis on the usage of icons and symbols. This suggestion supports previous research (68) which showed subtle messaging around foods was more impactful than explicit messaging, with adults more likely to select healthier items labelled with a heart logo than those with labels stating ‘a healthy choice’. Discussions with pupils and school staff also indicated the potential usage of colour-coded labels including traffic-light labels. A study conducted in secondary schools in Belgium found that increasing the number of healthier beverages available and applying a traffic-light labelling scheme to all items, effectively reduced adolescents’ consumption of sugar-sweetened beverages (SSB’s; labelled red) in both the school canteen and vending machine (69). Nonetheless, the importance of consultation with catering staff in individual schools prior to the design and implementation of labelling schemes was acknowledged in the present study to ensure feasibility with caterer’s daily routines, which aligns with previous research (70) reporting that school staff’s time constraints acts as one of the most dominant challenges to the implementation of school-based healthy lifestyle programmes.

Both pupil and school staff participants had a level of awareness of how adolescents’ food choices can be dependent on the location of items in the canteen, hence, manipulating the placement of the healthier options to more visible and accessible locations was a desirable concept among both stakeholder groups. Placement manipulations are a form of ‘nudge intervention’ which are generally minimal cost to implement (71). Implementing placement manipulations has resulted in increased and decreased purchases of fruit pots and SSB’s/sweet-baked goods, respectively, within United Kingdom secondary school canteens, albeit the evidence was limited (71). Further, school staff perceived items positioned first in the canteen serving area can directly impact adolescents’ food choices, and therefore, proposed serving the healthier items first as an effective strategy to improve food selection. This strategy has proven effective in breakfast buffet lines, with >75% of individuals opting for the first items they encountered (72), which could be easily transferred for implementation within school canteens.

The concept of receiving rewards to engage adolescents’ in healthy food-related behaviours in the school canteen has been positively viewed as a suitable strategy among socially deprived NI adolescents (aged 11–12 years) (30). Our findings in this study address a gap in the literature by further confirming the acceptability and feasibility of reward schemes (social, financial and recognition rewards) as incentives to promote positive food choices in school from the perspectives of both adolescents and school staff from a range of socio-economically diverse schools located in numerous district locations across NI. Tangible and praise rewards have previously proven effective in achieving positive food choice change by increasing fruit and vegetable consumption among elementary school children, although tangible rewards were more effective in the short and longer-term (73).

Overall, in line with a recent review (74), this qualitative study has identified various influential factors impacting adolescents’ food choices in secondary school canteens, with many also acting as barriers to the selection of the healthier food items in this setting. Moreover, similar to previous research with key school stakeholders outside the United Kingdom (75, 76). Participants in the present study perceived schools to be a viable setting to nurture healthy eating habits in adolescents and had clear ideas on practical and acceptable solutions, which could be implemented to better support adolescents making improved food choices in the school environment.

### Strengths and limitations

A strength of this study is that we successfully recruited a relatively large sample of mixed-gender pupils and staff in schools with socio-economically diverse profiles across a wide range of geographical locations in NI, including both urban and rural areas, increasing the generalisability of these findings. Given the complexity of adolescents’ dietary behaviours, the recruitment of adolescents, principals, vice-principals, teachers, caterers and EA staff enabled a holistic view of the primary factors influencing adolescents’ food choices within the school-setting and novel suggestions for improvement within this environment to be obtained, whilst also facilitating comparisons between a range of key stakeholder groups to be investigated. To our knowledge, this is the first study to explore NI school staff’s perspectives on adolescents’ school-based food choices and their recommendations for feasible intervention components to facilitate improvements in their dietary behaviours within this setting and to also compare these with the views of adolescents across NI. Moreover, schoolteachers in this study were recruited across a variety of subject disciplines, reducing the risk of selection bias of individuals particularly interested in the promotion of healthy eating within their school.

When interpreting the results, several limitations should be considered. It must be acknowledged that although a large sample of pupils of mixed-gender (*n* = 52 female; *n* = 34 male) were recruited to this study across 2-year groups, selection bias cannot be overlooked as only pupils who returned their study forms were eligible to be selected by a teacher for participation as the researcher (not affiliated with schools) was not involved in the selection process. Additionally, it is possible that pupils may have felt the need to provide desirable responses regarding their school’s food practises and their dietary behaviours, however, all pupils were informed by the researcher at the beginning of the focus groups that their responses would remain anonymous. Within the sample of pupils, 60% were female and 40% male, which is unlikely to introduce gender bias, however, it is important to note, that the participating school staff were predominantly female. Therefore, future work should consider achieving a gender-balanced sample by targeting male teachers within schools to encourage participation to determine if any gender differences may be present among school staff on this topic.

## Conclusion

Collectively, this research highlights the complexity of the multilevel factors which influence adolescents’ food choices within the school canteen and identifies barriers to achieving healthier dietary behaviours. Involving pupils and school staff to explore these barriers has highlighted a number of possible practical solutions to improve food choice in school, in particular those that are low-cost and non-labour intensive. Using the ecological framework, suggestions for improvement of food choices were identified at the individual (e.g., rewards), social (e.g., pupil-led initiatives), physical (e.g., labelling) and macro environment (e.g., whole-school approaches) level. At macro level, it is recommended that schools may wish to review the pricing policy to consider offering for example meal deals or special offers, with clear menu and labelling strategies to improve information for pupils at the point of purchase and also consider the location and placement of foods on sale. Additionally, it may be beneficial to implement a whole-school initiative, for example, healthy eating days/themes in the canteen to encourage all pupils to make healthier choices. This study highlights the importance of early consultation with school stakeholders to identify the existing influential factors on adolescents’ school-based food choices and which strategies are viewed as both acceptable to the target population and suitable for implementation within the school-setting by key school staff members which should be considered in future intervention design. Further research is needed to determine the feasibility of implementing these intervention strategies within the school-setting and to test their effectiveness in practice.

## Data availability statement

The raw data supporting the conclusions of this article will be made available by the authors, without undue reservation.

## Ethics statement

The studies involving human participants were reviewed and approved by Ulster University Research Ethics committee FCBMS-20-016-A; REC/20/0031. Written informed consent to participate in this study was provided by the participants' legal guardian/next of kin.

## Author contributions

LD, AH, and AG: conceptualisation, data coding, data analysis, and interpretation of findings. LD, AH, SB, and AG: methodology. LD and SB: recruitment. LD conducted the interviews and focus groups and drafted the manuscript. AH, SB, and AG critically reviewed the manuscript. All authors contributed to the article and approved the submitted version.

## Funding

This study was undertaken as part of a PhD scholarship (LD) funded by the Department for the Economy (DfE).

## Conflict of interest

The authors declare that the research was conducted in the absence of any commercial or financial relationships that could be construed as a potential conflict of interest.

## Publisher’s note

All claims expressed in this article are solely those of the authors and do not necessarily represent those of their affiliated organizations, or those of the publisher, the editors and the reviewers. Any product that may be evaluated in this article, or claim that may be made by its manufacturer, is not guaranteed or endorsed by the publisher.
